# Paraganglioma of the spermatic cord: A case report and literature review

**DOI:** 10.1016/j.ijscr.2019.06.047

**Published:** 2019-06-26

**Authors:** Brendan Gontarz, Poornima Hegde, David McFadden

**Affiliations:** a Department of Surgery, University of Connecticut, 263 Farmington Avenue, Farmington, CT, 06032, United States; b Department of Pathology and Laboratory Medicine, University of Connecticut, 263 Farmington Avenue, Farmington, CT, 06032, United States

**Keywords:** Paraganglioma, Pheochromocytoma, Spermatic cord, Case report

## Abstract

**Introduction:**

Neuroendocrine tumors are a diverse and well-described entity with most arising from the gastrointestinal tract. A clinically significant example is the paraganglioma, or an extra-adrenal catecholamine-producing tumor.

**Presentation of case:**

Herein we describe and review a paraganglioma arising from the spermatic cord in a 55 year old asymptomatic man.

**Discussion:**

Paragangliomas are rare, with an incidence of 3–8 cases per million population. To date there are only 12 cases found in the literature.

**Conclusion:**

In this article, we review our patient’s presentation, follow-up, and screening followed by a review of the literature of this fascinating tumor. Although rare, paraganglioma should be considered in the differential diagnosis of unusual groin masses. This work has been reported in line with the SCARE criteria (Agha et al., 2018).

## Highlights

• A paraganglioma, or an extra-adrenal catecholamine-producing tumor, is a clinically significant neuroendocrine tumor with an incidence of 3–8 cases per million population.
• The majority of paragangliomas, 85–90%, occur in the adrenal glands and 98% are found in the abdomen.
• Unlike pheochromocytomas, where only 10% are malignant, paragangliomas carry a 40–50% malignancy rare, and their development is part of a hereditary syndrome in 30% of cases.
• The most common hereditary syndromes associated with paragangliomas are von Hipple-Lindau (VHL), multiple endocrine neoplasia 2 (MEN 2) and neurofibromatosis type 1 (NF1).
• Herein we describe and review the 13th paraganglioma documented arising from the spermatic cord.

## 1 Case presentation

A 55 year-old man sought surgical excision of a left sided inguinal mass. This mass had been present for approximately 1 year and was firm and non-tender. When seen in the surgical office, the differential diagnosis included hernia, lymphadenopathy, or inguinal cyst. An ultrasound was performed and demonstrated a well-defined 2.5 cm × 1.8 cm × 2.0 cm solid left inguinal mass with areas of central cystic change. The findings were considered non-specific and thought to represent a necrotic lymph node or soft tissue mass. Tissue diagnosis was recommended [1].

The patient did have a past medical history of an episode of palpitations and light headedness the previous year. He was diagnosed with a non-ST elevated myocardial infarction. A subsequent nuclear stress test and were normal. He was not hypertensive. His family history was positive for a father who died of colon cancer in his 80 s but no other cancers or known history of neuroendocrine abnormalities.

The patient was brought to the operating room and under general anesthesia a standard oblique groin incision was made. A rubbery, well defined 3 cm mass was noted associated with the spermatic cord. The mass was well vascularized and excised without sacrificing cord structures. It was noted that the patient became very hypertensive during the manipulation of the mass; however this resolved once the mass was removed. The remainder of the case was uneventful and the patient was discharged from the recovery room.

**Fig. 1:**
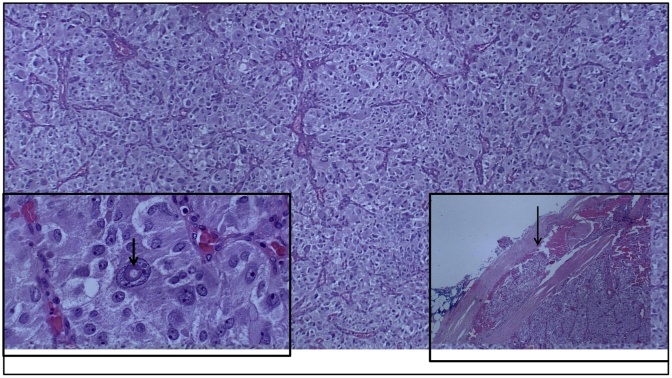
Paraganglioma with organoid/nested (Zallballen) pattern; nests separated by vascularized, thin fibrous septa (H&E, original magnification ×40). Lower left inset: Large polygonal tumor cells with granular cytoplasm, pleomorphic and bizarre nuclei with prominent nucleoli; occasional (arrow) intranuclear inclusions are present (H&E, original magnification ×400). Lower right inset: Vascular invasion (arrow) in paraganglioma (H&E, original magnification ×40).

**Fig. 2:**
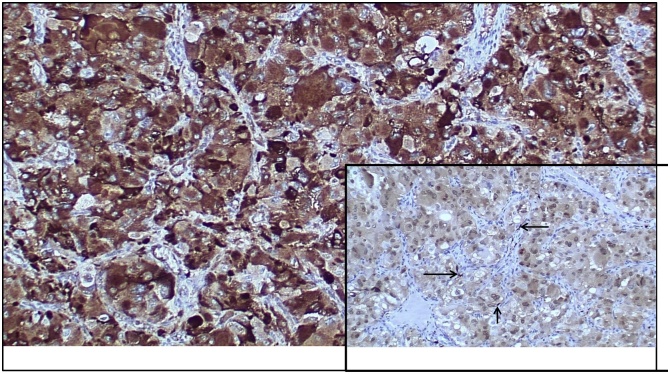
Tumor cells with diffuse granular cytoplasmic staining for antibody to chromogranin (immunohistochemical stain, original magnification ×200). Lower right inset: Occasional (arrow) sustentacular cells at the periphery of tumor cell nests, highlighted by antibody to S-100 (immunohistochemical stain, original magnification ×100).

The pathologic evaluation revealed a 2.5 cm paraganglioma that was completely excised; a rare focus of vascular invasion was noted (Fig. 1). The mass had large polygonal tumor cells with granular cytoplasm and pleomorphic to bizarre nuclei, with rare intranuclear inclusions (Fig. 1). The mitotic rate was low at 0-3/10 high power fields. The tumor cells showed diffuse and strong immunoreactivity for antibodies to chromogranin, synaptophysin and neuron specific enolase (NSE); rare sustentacular cells were highlighted by S-100 immunostain (Fig. 2). Cells stained negatively for vimentin, epithelial membrane antigen (EMA) and GATA-3. Given these findings the patient was referred to an endocrinologist for further work up. On physical exam he did have 3 additional subcutaneous nodules on his leg and chest. He had no café au lait spots. He was screened with a thyroid function panel, plasma metanephrines, testosterone, lutenizing hormone, follicular stimulation hormone, and calcitonin; all were all within normal limits. He also underwent an ultrasound examination of his neck and thyroid, as well as a CT scan of his abdomen and pelvis along with a separate scan specifically focusing on the pancreas and adrenal glands. The only findings were a small 5 mm right adrenal nodule that appeared to be a benign adenoma. He subsequently refused genetic testing. Two years later the patient is doing well, the aforementioned soft tissue masses were excised at the patient’s request, and were all benign lipomas.

## 2 Discussion

Paragangliomas arise from chromaffin cells, which are neuroendocrine cells found predominantly in the adrenal medulla. The terminology for these tumors is dependent on the location of the tumor. If present in the adrenal medulla it is a pheochromocytoma. If located outside the adrenal gland they are called sympathetic paraganglioma [3]. Embryologically they arise from the neural crest cells that later in development become the chromaffin cells. These cells migrate along the sympathetic ganglia in the paravertebral axis and settle in the adrenal medulla [4]. The second most common location is the Organ of Zuckerkandl at the bifurcation of the aorta or origin of the inferior mesenteric artery. Chromaffin cells are also found in the vagus nerve, carotid bodies, bladder wall, prostate and behind the liver [5]. The majority, 85–90%, of tumors occur in the adrenal glands and 98% are found in the abdomen [3]. Despite differences in location, tumors arising from the paraganglion system are morphologically similar to each other.

To date there are only 12 cases in the medical literature in which a paraganglioma was found in the spermatic cord [[5], [6], [7], [8], [9], [10], [11], [12], [13], [14], [15], [16]]. The most recent report [14] was the first that displayed a malignant phenotype. Unlike pheochromocytomas, where only 10% are malignant, paragangliomas carry a 40–50% malignancy rate [3,4]. In paragangliomas, histomorphologic features such as pleomorphic/bizarre nuclei and vascular invasion, as were seen in our case, are not to be taken as evidence of malignancy. The *sine qua non* of malignancy is evidence of metastasis.

The development of these tumors is thought to be part of a hereditary syndrome in 30% of cases. The most common are von Hipple-Lindau (VHL), multiple endocrine neoplasia 2 (MEN 2) and neurofibromatosis type 1 (NF1). There have been 10 identified gene sites where mutation has led to the development of these tumors [3]. Although exceedingly rare, this should be a diagnostic possibility that surgeons and urologists are aware of given the extremely common operation of inguinal hernia repairs. The possibility of incidentally coming across a mass in the spermatic cord and regarding it as a mere cord lipoma, without proper examination by pathology, could lead to a missed diagnosis and further complications. Given the common heredity of these tumors and the possibility for other primary tumors or metastasis [12,16], patients warrant a prompt work up by a neuroendocrinologist and may even warrant genetic counseling.

In our patient, it is unclear whether this disease could have been identified earlier given his one prior episode of palpitations and lightheadedness. It is unlikely given that he only had symptoms once; if there were multiple occurrences in the classical “episodic” pattern, suspicion would have been raised. The patient had this mass identified, properly excised, and upon review of the pathology was referred to the appropriate services for further evaluation. He completed a thorough evaluation for other primary, metastatic, or hereditary diseases, and all were negative. An unrecognized potential danger was the surgery itself as the patient did become hypertensive in the operating room as the mass was manipulated. The standard for a known catecholamine secreting tumor is alpha and possible beta blockade prior to any resection or biopsy. Surgeons should be aware of this rare possibility.

If diagnosed postoperatively, evaluation includes biochemical marker assays: plasma metanephrines, thyroid function tests, prostate specific antigen, cancer antigen 19-9, luteinizing hormone, follicular stimulating hormone, testosterone and calcitonin [3,12,16]. These laboratory values assess for distant disease, other primaries as part of a hereditary syndrome, or metastasis. Imaging should also be done with computed tomographic (CT) scan being the preferred modality to evaluate the pancreas and adrenals [6]. Ultrasound is useful to evaluate the neck, carotid bodies and thyroid [3]. Suspicious findings may warrant an octreotide scan [7].

On review of the previous 12 cases identified, only four were identified to have hormonal activity [7,12,15,16]. Two of the cases had other tumors noted on their work up [12,14]. In one case, the patient had bilateral carotid body tumors and bilateral pheochromocytomas surgically removed. Five years later a recurrence was found, which presented as a left scrotal mass [12]. The other case with a second tumor was the first reported “giant” paraganglioma that had a left paraspinal lung metastasis [14]. There is no report in the literature of any intraoperative hypertensive complications; a minority of paragangliomas express catecholamines systemically [5].

We would classify our patient as having hormonal activity given the intra-operative hypertension and cardiac symptoms the year prior.

Lifelong follow up is recommended [16]. Amar, et al, recommend measuring urine and plasma metanephrines 1 month after surgery [16]. In patients with sporadic, single tumors ≤5 cm in diameter, follow up for symptoms and monitoring with blood chemistries can be done every two years. Patients with larger or multiple but apparently benign tumors or inherited disease should be tested at least every 6 months after surgery and then every year lifelong. Imaging follow-up is also required in patients with inherited or malignant tumors although specific timing varies based on disease [16]. Management and follow up ultimately leads to the best outcomes for the patients and early detection of recurrence.

## 3 Conclusion

Paragangliomas of the spermatic cord are a rare entity that should be recognized by surgeons and urologists. Diagnosis is rarely made preoperatively. Successful management requires pre-operative biochemical evaluation when possible, followed by surgical resection.

## Sources of funding

Canzonetti U Conn Foundation Fund will pay the $1000.0 if accepted for publication.

## Ethical approval

University of Connecticut IRB exempts de-identified case reports and brief Case series from review.

## Consent

Written consent from the patient is confirmed and available upon request.

## Author’s contribution

Brendan Gontarz, MD: data collection, writing the paper.

Poornima Hegde, MD: data collection, data analysis or interpretation.

David McFadden, MD: study concept or design, data collection, writing the paper.

## Registration of research studies

N/A.

## Guarantor

David McFadden, MD.

## Provenance and peer review

Not commissioned, externally peer-reviewed

## Declaration of Competing Interest

None.
